# Role of glucocorticoid-induced leucine zipper (GILZ) in inflammatory bone loss

**DOI:** 10.1371/journal.pone.0181133

**Published:** 2017-08-03

**Authors:** Nianlan Yang, Babak Baban, Carlos M. Isales, Xing-Ming Shi

**Affiliations:** 1 Departments of Neuroscience & Regenerative Medicine, Augusta University, Augusta, GA, United States of America; 2 Departments of Oral Biology, Augusta University, Augusta, GA, United States of America; 3 Departments of Orthopaedic Surgery, Augusta University, Augusta, GA, United States of America; Universite de Nantes, FRANCE

## Abstract

TNF-α plays a key role in the development of rheumatoid arthritis (RA) and inflammatory bone loss. Unfortunately, treatment of RA with anti-inflammatory glucocorticoids (GCs) also causes bone loss resulting in osteoporosis. Our previous studies showed that overexpression of glucocorticoid-induced leucine zipper (GILZ), a mediator of GC’s anti-inflammatory effect, can enhance osteogenic differentiation *in vitro* and bone acquisition *in vivo*. To investigate whether GILZ could antagonize TNF-α-induced arthritic inflammation and protect bone in mice, we generated a TNF-α-GILZ double transgenic mouse line (TNF-GILZ Tg) by crossbreeding a TNF-α Tg mouse, which ubiquitously expresses human TNF-α, with a GILZ Tg mouse, which expresses mouse GILZ under the control of a 3.6kb rat type I collagen promoter fragment. Results showed that overexpression of GILZ in bone marrow mesenchymal stem/progenitor cells protected mice from TNF-α-induced inflammatory bone loss and improved bone integrity (TNF-GILZ double Tg vs. TNF-αTg, n = 12–15). However, mesenchymal cell lineage restricted GILZ expression had limited effects on TNF-α-induced arthritic inflammation as indicated by clinical scores and serum levels of inflammatory cytokines and chemokines.

## Introduction

Chronic inflammatory conditions such as rheumatoid arthritis (RA) and inflammatory bowel disease are known to cause bone loss [1, 2]. Tumor necrosis factor alpha (TNF-α), is a potent proinflammatory cytokine and plays a major pathogenic role in RA [3–5]. TNF-α induces inflammation by activating NF-kB and AP-1, two key inflammatory mediators that activate the transcription of an array of inflammatory genes including cyclooxygenase-2 (COX-2) [6, 7], a target of a class of anti-arthritis medications, COXIBs such as celecoxib, etoricoxib and rofecoxib. Chronic inflammation is known to result in bone loss [8–10]. Ironically, medications used to suppress inflammation such as glucocorticoids (GCs), also cause bone loss, making an already devastating condition, e.g., rheumatoid arthritis (RA), even worse. TNF-α inhibitors such as etanercept, infliximab, adalimumab, certolizumab pegol, and golimumab, have become a cornerstone in RA therapy because of their effectiveness in suppressing inflammation and protecting bone [11–15]. However, these medications have many side effects including malignancies [16, 17] and cardiovascular events [17–19], among others [17, 20–25].

High doses of GCs have been used for decades in the treatment of autoimmune disorders such as RA [26, 27]. However, their use results in osteoporosis (GC-induced osteoporosis, GIOP) in half of the affected patients [28, 29]. Intestingly, low-dose or physiological concentrations of GC may have bone protective effects in RA patients [30]. Glucocorticoid-induced leucine zipper (GILZ) is a GC-inducible gene [31] and has been demonstrated to be an anti-inflammatory effect mediator for GCs [32–34]. We previously showed that overexpression of GILZ in bone marrow mesenchymal stem cells (MSCs) can enhance osteogenic differentiation [35], antagonize the inhibitory effect of the inflammatory cytokine TNF-α on MSC osteogenic differentiation [36], and enhance bone acquisition in mice [37]. These data suggested that GILZ mediates not only GC’s anti-inflammatory effect but also bone anabolic actions, making it a potential candidate for new anti-inflammatory therapies. In this study, we sought to investigate whether GILZ overexpression could antagonize TNF-α-induced arthritic inflammation and protect bone by using a TNF-α transgenic mouse, an animal model of RA that spontaneously develops polyarthritis.

## Materials and methods

### Chemicals and antibodies

All chemicals were purchased from Thermo Fisher Scientific (Pittsburgh, PA, USA) or Sigma-Aldrich (St. Louis, MO, USA) except where specified.

### Animals

TNF-α transgenic (TNF-α Tg) mice overexpressing human the TNF-α gene were provided by Dr. George Kollias [38]. The GILZ Tg mice used, in which the expression of mouse GILZ gene is under the control of a 3.6kb rat type I collagen promoter (Col3.6), were previously described [37]. Both GILZ and TNF-α Tg mice were on a C57BL background. To generate TNF-GILZ double Tg mice, female TNF Tg mice were backcrossed with male GILZ Tg mice. Most experiments were carried out using sex matched littermates except where a relatively larger number of mice are needed we pooled both males and females together to meet the statistical requirement as no sex difference was noted in our previous study [37]. All animal procedures were performed in accordance with a protocol (#2008–0302) approved by the Augusta University Institutional Animal Care and Use Committee. Mice were housed in a barrier facility at the Laboratory Animal Service facility under a 12-hr dark-light cycle and fed with standard rodent chow and water ad libitum. Mice were euthanized using CO_2_ overdose followed by thoracotomy according to AU-IACUC approved animal protocols.

All mice were evaluated weekly for signs of arthritis at the age of 7 weeks in a blinded fashion. A clinical score was assigned to each ankle joint using a semi-quantitative scoring system [39]: 0 for no arthritis, 1 for mild arthritis (joint swelling), 2 for moderate arthritis (severe joint swelling and deformity), and 3 for severe arthritis with motion limitation. The total mouse clinical score was the sum of the scores from all four ankles.

### DXA and μ-CT analyses

Ten to twelve mice from each experimental group (GILZ Tg, TNF Tg, and TNF-GILZ Tg) were assessed for bone density and architecture. The left femur of each mouse was dissected free of soft tissues and scanned by DXA densitometry (Lunar PIXImus II mouse densitometer; GE Medical Systems) for bone mineral density (BMD) and bone mineral content (BMC) measurement. Bone structural parameters of the right femur and tibia of each mouse were analyzed by μ-CT scan (μ-CT-40; Scanco Medical AG) as previously described [37].

### Histology and immunohistochemistry analyses

After DXA scan, the left femur and ankle of each mouse were decalcified in EDTA, embedded in paraffin and sectioned at 4–6 μm for histomorphometry. Sections were stained with hematoxylin and eosin for calculation of osteoblast numbers. A Bioquant Osteo image analysis system (Bioquant, Nashville, TN) was used to count the number of osteoblasts and measure the osteoblast covered areas. A defined region of interest was established ~0.5mm proximal to the distal growth plate and extended a further 0.5mm, all within the endocortical edges at 40x magnification.

For immunohistochemistry, paraffin sections of femurs were deparaffinized in xylene and rehydrated by passing the slides through graded alcohol solutions. Endogenous peroxidase was quenched with 3% H_2_O_2_ in PBS. The sections were then washed in distilled water and heated at 95°C in antigen retrieval buffer (Dako, Glostrup, Denmark). Nonspecific staining was blocked with 5% normal goat serum in PBS for 1 h. Endogenous biotin was inhibited with an avidin/biotin blocking kit (SP2001, Vector Laboratories, Burlingame, CA). The sections were then incubated with an anti-osteocalcin polyclonal antibody (PB9919, Boster Bio, Pleasanton, CA) diluted at 1:1000 in 2% normal goat serum overnight at 4°C. Next, the sections were incubated with biotinylated goat anti-mouse/rabbit antibody (1:1,000) (Jackson ImmunoResearch Laboratories, West Grove, PA) for 30 min. Sections were then washed and incubated with StreptABComplex/HRP (Dako) for 45 min. Antibody binding was revealed using a DAB kit (SK4100, Vector Laboratories). Negative controls were obtained by omission of the primary antibody. Counterstaining was performed with Mayer's hematoxylin (Sigma).

### Isolation and culture of synovial fibroblasts

Synovial fibroblasts were isolated from the paws of both GILZ and TNF-α Tg mice according to the protocol of Armaka et al. [40]. Briefly, paw joints were dissected and cleared free of skin and soft tissues, then digested in 1mg/ml collagenase IV at 37°C for 1h. Cells were released via vigorous vortex and cell suspension were seeded into cell culture dishes and incubated in a humidified tissue culture incubator. DMEM supplemented with 10% FBS, 1% L-Glutamine and 1% pen/strep was used to culture cells and the medium was replaced every 3 days. Cells were used before passage 4. Three to four mice were used in each group and the experiment was repeated twice.

### RNA extraction and real-time qRT-PCR

Total cellular RNA was isolated using TRIzol reagent following manufacturer's instructions (Invitrogen Corporation, Grand Island, NY, USA). Equal amounts of total RNA (2 μg) were reverse transcribed using iScript cDNA synthesis kit (Bio-Rad, Hercules, CA, USA) and the mRNA levels of the indicated genes were analyzed in triplicate using SYBR Green Master Mix and a Chromo-4 real-time RT-PCR instrument. The mRNA levels were normalized to β-actin (internal control), and gene expression was presented as fold change. The primer sequences used in the PCR reactions were: GILZ (AF024519): 5′-GCTGCACAATTTCTCCACCT-3′ (forward) and 5′-GCTCACGAATCTGCTCCTTT-3′ (reverse); β-actin (NM_007393): 5′-CTGGCACCACACCTTCTACA-3′ (forward) and 5′-GGTACGACCAGAGGCATACA-3′ (reverse); IL-6 (NM_031168.2): 5’-GCCAGAGTCCTTCAGAGAGATACA-3’ (forward) and 5’-CTTGGTCCTTAGCCACTCCTTC-3’ (reverse).

### Flow cytometry

Three to four mice were used in each group and the experiment was repeated twice. Both tibiae and femur were dissected free of soft tissues and bone marrow cells flushed with RPMI 1640 medium supplemented with 10% FBS. Blood was collected by cardiac puncture. Cells were first incubated with antibodies against cell surface markers CD11b, and then fixed and permeabilized using Fixation/Permeabilization Concentrate (Cat. No.00-5123-43, eBioScience) before incubation with antibodies for intracellular labeling of human TNF-α (BD BioSciences, Bedford, MA, USA). After one wash, cells were run through a 4-color flow cytometer (FACSCalibur, BD Biosciences, San Diego, CA, USA), and data were collected using CellQuest software (BD Biosciences, San Jose, CA, USA) as previously described [25]. As a gating strategy, for each sample, isotype-matched controls were analyzed to set the appropriate gates. For each marker, samples were analyzed in duplicate measurements. To minimize false-positive events, the number of double-positive events detected with the isotype controls was subtracted from the number of double-positive cells stained with corresponding antibodies (not isotype control), respectively. Cells expressing a specific marker were reported as a percentage of the number of gated events. Statistical analysis was performed using Prism 5.0 software (GraphPad Software, Inc. La Jolla, CA, USA).

### Serum assays

Blood samples were centrifuged at 2,000 rpm for 10 min at 4°C and the serum collected and stored at −80°C in aliquots. Receptor activator of nuclear factor kappa-B ligand (RANKL) and osteoprotegerin (OPG) levels were measured using Quantikine ELISA Kits (Cat No: MTR00 and MOP00, respectively, R&D Systems). Serum concentrations of selected cytokines and chemokines were measured using a fluorescent bead-based Multiplex immunoassay on a Luminex 200 machine (Luminex Corporation, Austin, TX). Serum collected from TNF Tg and TNF-GILZ Tg mice (12–15 in each group, age and sex matched) were analyzed. All samples were assayed in duplicate.

### Statistical analyses

The results are expressed as means ± S.D. The data were analyzed using either analysis of variance with Bonferroni post hoc testing or unpaired *t* tests, using Prism 5.0 software. A *p* value less than 0.05 is considered significant.

## Results

### Effect of GILZ on TNF-α-induced arthritis

**Fig 1:**
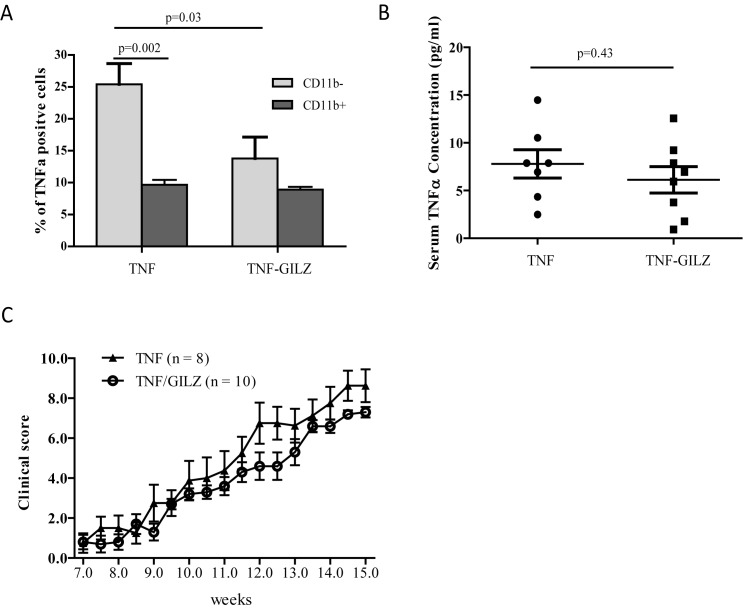
Characterization of TNF-GILZ Tg mice. (**A**) FACS analysis showing percentages of human TNF-α-positive hematopoietic (CD11b+) and mesenchymal (CD11b-) lineage cell populations in bone marrow of the TNF- α and TNF-GILZ double Tg mice. Three to four mice were used in each group and the experiment was repeated twice with similar results. (**B**) ELISA assays showing serum levels of mouse TNF-α in TNF and TNF-GILZ Tg mice. Each data point represents one mouse. (**C**) Clinical arthritis scores of TNF and TNF-GILZ Tg mice. The results are expressed as means ± S.D. Unpaired t-tests were performed for comparison.

To test whether GILZ can offset or reduce the degree of TNF-α-induced arthritic inflammation, we created TNF-GILZ double transgenic (TNF-GILZ Tg) mice by crossbreeding GILZ Tg mice with TNF-α Tg mice. The TNF-α Tg mouse carries a human TNF-α gene and is expressed ubiquitously [41]. This mouse develops spontaneous polyarthritis at ~7 weeks of age. The GILZ Tg mouse bears a mouse GILZ gene under the control of a 3.6kb rat type I collagen promoter fragment (Col3.6), thus its expression is restricted to bone marrow mesenchymal lineage cells (MSCs) and not in hematopoietic lineage cells (BMMs) as we have previously shown [42]. FACS analysis of whole bone marrow cells confirmed that both the TNF-α Tg and TNF-GILZ double Tg mice have high percentages of cells positive for human TNF-α in both mesenchymal (CD11b-) and hematopoietic (CD11b+) lineage cell populations (Fig 1A). Although the percentage of TNF-α-positive cells was reduced in mesenchymal lineage cells, serum levels of mouse TNF-α remained unchanged in TNF-GILZ double Tg mice compared with that in the TNF-α Tg mice (Fig 1B). Similar to the TNF-α Tg mice, which begin to develop spontaneous polyarthritis at the age of 7 weeks as previously reported [41], the TNF-GILZ double Tg mice also started to develop polyarthritis (Fig 1C), indicating that mesenchymal lineage cell expression of GILZ does not counteract or delay the development of TNF-α -induced arthritis in mice expressing high levels of human TNF-α globally.

### Overexpression of GILZ protects bone from TNF-α-induced destruction

**Fig 2:**
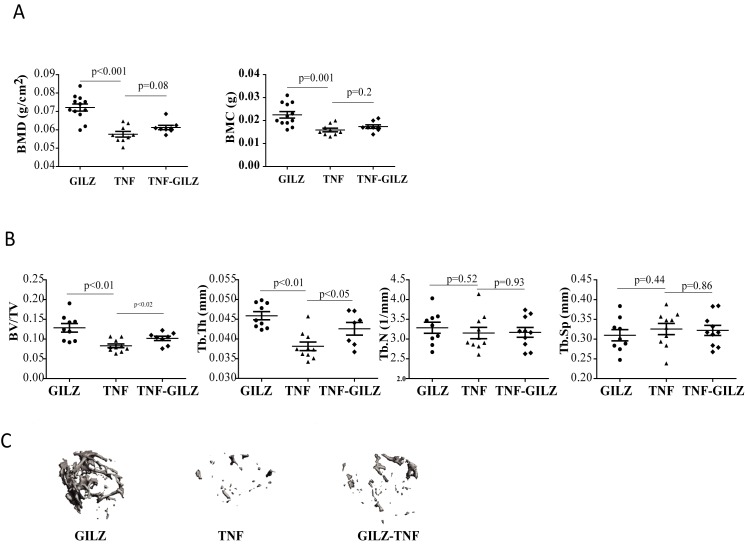
Bone phenotype of TNF-GILZ Tg mice. (**A**) DXA analysis of femoral samples showing bone mineral density (BMD) and content (BMC) of 6-month-old mice. Each data point represents one individual mouse. (**B**) uCT analysis showing bone integrity and architecture parameters of TNF and TNF-GILZ Tg mice. (**C**) Representative re-constructed 3D uCT images (femurs) of TNF and TNF-GILZ Tg mice. Samples of GILZ Tg littermates were used as a reference for GILZ anti-TNF-α actions. Results are expressed as means ± S.D. ANOVA with Bonferroni post hoc tests were performed for comparison.

**Fig 3:**
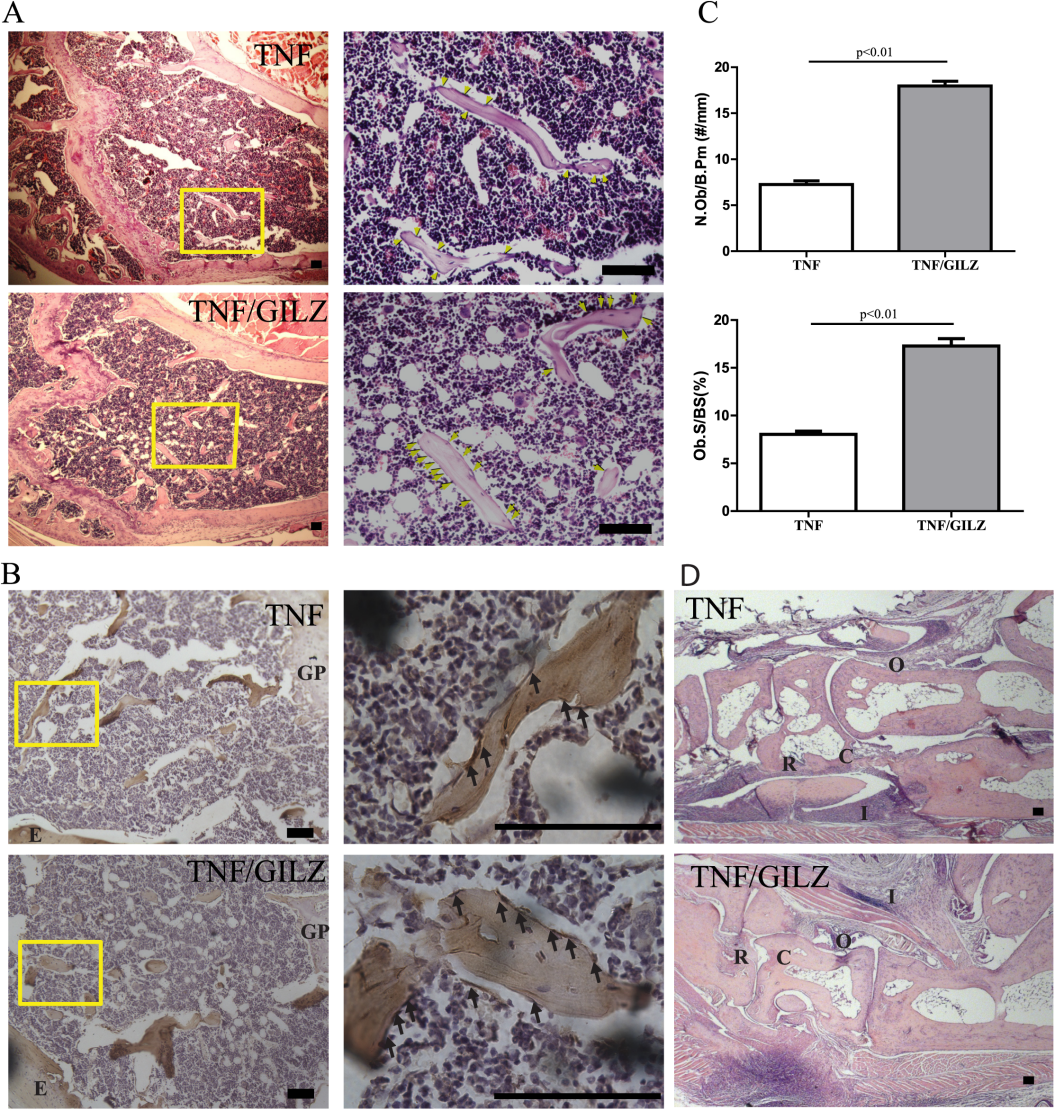
H&E histology and histomorphormetry analysis of bone and joint. (**A**) Representative images of H&E stained femur of TNF and TNF-GILZ Tg mice (enlarged boxed areas are shown on right). (**B**) Representative IHC staining images of femurs. Boxed areas are enlarged (right) and arrows indicate osteocalcin-positive osteoblast cells. GP: growth plate; E: endocortical bone. (**C**) Bar graph showing quantified results of A. A Bioquant osteo image analysis system (Bioquant, Nashville, TN) was used to count the number of osteoblasts and measure the oasteoblast covered area. A defined region of interest was established ~0.5mm proximal to the distal growth plate and extended a further 0.5mm, all within the endocortical edges at 50x magnification. A total of 4 to 5 samples were analyzed for each group. (**D**) Representative histological images of paw-joint of TNF and TNF-GILZ Tg mice (20x). C: Cartilage damage; R: Bone resorption; I: inflammation; O: Osteophyte. Scale bar = 100μm.

We then examined whether GILZ can protect TNF-α-induced inflammatory bone loss. DXA analysis showed that both the bone mineral density (BMD) and bone mineral content (BMC) exhibit a trend of increased mineralization in TNF-GILZ mice when compared with TNF-α mice, although these increases were not statistically significant (Fig 2A). However, μCT analysis showed significant increases in bone volume density (BV/TV) and trabecular thickness (Tb.Th) in double Tg mice when compared with the TNF-α mice (Fig 2B). No difference were detected in trabecular number (Tb.N) or trabecular spacing (Tb.Sp). Representative re-constructed 3D images of trabecular bone are shown (Fig 2C). Histology and histomorphometry studies of decalcified femur and joint samples showed that TNF-GILZ double Tg mice have increased osteoblast numbers (Ob.N) and osteoblast covered surface areas (Ob.S/BS) compared to that in the TNF-α Tg mice (Fig 3A and 3C). The same results were obtained by immunohistochemical staining of slides with anti-osteocalcin antibody (Fig 3B). Consistent with the clinical scoring data (Fig 1C), histological analysis of the paw joint sections showed no significant difference in either inflammation or joint structure between TNF-α Tg and TNF-GILZ double Tg mice (Fig 3D). Together, these results indicated that overexpression of GILZ in MSCs can protect or preserve, to some degree, trabecular bone integrity from inflammatory destruction.

### GILZ modulates TNF-α-induced serum levels of cytokines and chemokines

**Fig 4:**
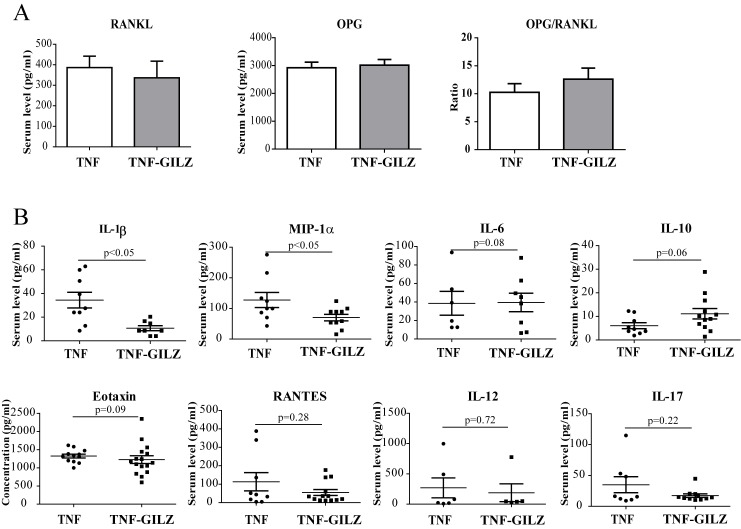
Serum cytokine and chemokine levels in TNF and TNF-GILZ Tg mice. (**A**) ELISA assay showing serum levels of RANKL and OPG, and the ration of OPG/RANKL in TNF and TNF-GILZ Tg mice. Six to eight mice were used in each group. The experiment was repeated twice with similar results. (**B**) Multiplex immunoassay showing serum levels of different cytokines and chemokines in TNF and TNF-GILZ Tg mice. Each data point is from one individual mouse.

To determine if the inflammatory profile was altered in TNF-GILZ double Tg mice, we measured serum levels of osteoprotegerin (OPG) and receptor activator of nuclear factor kappa-B ligand (RANKL), two factors that play important roles in bone metabolism, and several inflammatory cytokines and chemokines that are involved in arthritic inflammation and bone loss [4, 43–47]. Results showed that serum levels of OPG and RANKL were not significantly different between TNF-GILZ double Tg mice and TNF-α Tg mice (Fig 4A). Serum levels of IL-1β and MIP-1α were significantly lowered in TNF-GILZ double Tg compared with that in TNF-α Tg mice (Fig 4B). Levels of IL-6 were decreased while IL-10 were increased in TNF-GILZ double Tg mice although these changes were not statistically significant (p = 0.08 and 0.06, respectively). No significant difference was detected between TNF-GILZ double Tg and TNF-α Tg mice in levels of other cytokines and chemokines examined including eotaxin, RANTES, IL-12, and IL-17 (Fig 4B).

**Fig 5:**
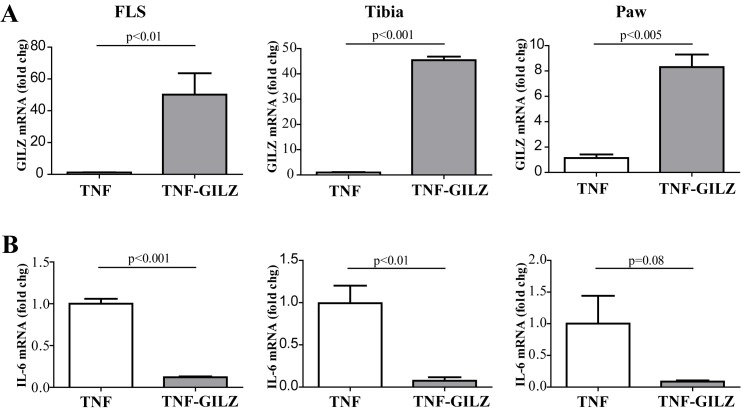
GILZ and IL-6 expression in synoviocytes and tibia and paw tissues. Real time qRT-PCR analysis showing levels of GILZ (**A**) and IL-6 mRNA (**B**) in fibroblast like synoviocytes (FLS) and tibia and paw tissues as indicated. Data is presented as fold change. Value from TNF mice is arbitrarily set as 1. Three to four mice were used in each group and the experiment was repeated twice with similar results.

Finally, we examined the local effects of GILZ in bones and joints, as well as in fibroblast-like synoviocytes (FLS) since these cells are considered bona fide MSCs [48]. High levels of GILZ mRNA were detected in FLS cells isolated from TNF-GILZ double Tg mice compared with the TNF-α Tg mice (Fig 5A). The same mRNA expression pattern was also observed in RNA samples isolated from tibiae and paw tissues (Fig 5A). Accordingly, the mRNA levels of IL-6 were reduced significantly in RNA samples isolated from FLS cells and bone tissues (tibia and paw) of the TNF-GILZ double Tg mice (Fig 5B).

## Discussion

Rheumatoid arthritis (RA) is a chronic inflammatory disease affecting not only joints but also the skeleton leading to systemic bone loss and increased risk of fractures. TNF-α is one of the key factors responsible for inflammation and bone loss in RA [13]. Studies carried in animal models have shown that TNF-α promotes the differentiation of bone resorbing osteoclasts while inhibiting the activity of bone forming osteoblasts [13, 49, 50]. Clinical evidence show that inhibition of TNF-α is effective in suppressing inflammation but reports on the effectiveness of TNF inhibition in preventing bone loss are inconsistent [11, 12, 51, 52]. Thus, a search for new therapies is necessary.

Glucocorticoids (GCs) are among the most potent anti-inflammatory drugs for treatment of RA, but their clinical usage is limited due to their severe side effects on bone (GC-induced osteoporosis) [12, 28, 53, 54]. Compelling evidence show that glucocorticoid-induced leucine zipper (GILZ) protein is a GC anti-inflammatory effect mediator [32, 33, 55]. We previously showed that overexpression of GILZ can enhance MSC osteogenic differentiation *in vitro* and it promotes bone acquisition in mice [35, 37], Furthermore, we found that overexpression of GILZ inhibits inflammatory cytokine-induced COX-2 expression [32] and antagonizes the inhibitory effect of TNF- α on MSC osteogenic differentiation *in vitro* [36]. These studies prompted us to investigate whether GILZ is capable of counteracting TNF-α-elicited arthritic inflammation thus protect against systemic bone loss in TNF-α Tg mice. Data presented in this study showed that overexpression of GILZ can protect against bone loss, which is consistent with our previous finding [35, 37], but its effects on arthritic inflammation are limited. Also, the TNF-GILZ Tg mice did not display a significant improvement on bone erosion or joint damage compared with TNF Tg mice (Fig 3C), confirming our previous report that overexpression of GILZ in MSC/progenitor cells does not have an effect on osteoclast differentiation or activity [37].

Interestingly, we noticed that local expression of IL-6 in bone and joint, and in FLS is significantly reduced in TNF-GILZ double Tg mice compared to that in TNF-αTg mice. This seemed unlikely since the FLS cells reside in joint which is outside of bone marrow cavity and should not overexpress GILZ in TNF-GILZ mice. However, there are evidence showing that the onset of RA is associated with a massive influx of mesenchymal cells into the joint [56, 57]. These mesenchymal cells or FLS are identified as bona fide bone marrow MSCs. These MSCs are recruited to the arthritic joints but, due to the inflammation, their normal differentiation is arrested and they acquire a ‘‘tumor-like” phenotype and are thought to play a key role in the pathogenesis of RA [58]. Our data showed that GILZ is expressed in these cells (Fig 5A) and suppresses the expression of IL-6 (Fig 5B). Both IL-6 and TNF-α are recognized as key cytokines in RA pathogenesis and, a recent study by Malysheva et al suggested that IL-6 blockade could partially rescue osteogenesis from the negative effect of TNF-α via Wnt signaling pathway [59]. Therefore, GILZ inhibition of IL-6 may explain, at least in part, its protective effect from inflammatory bone loss. We were unable to detect a reduction of serum IL-6 in TNF-GILZ mice. The main reason for this discrepancy, we think, is that the GILZ transgene expression is restricted to mesenchymal lineage cells (MSCs, osteoblasts and adipocytes) but TNF-α is overexpressed systemically in these mice. Indeed, the production of inflammatory cytokines by hematopoietic lineage cells (CD11b-positive cells) was not affected in TNF-GILZ double Tg mice (Fig 1A and 1B). This may also explain why GILZ, a GC anti-inflammatory effect mediator, failed to suppress inflammation in TNF-GILZ double Tg mice. It is expected that the anti-inflammatory effect of GILZ would be much stronger if GILZ were overexpressed globally or in hematopoietic lineage cells such as macrophages/neutrophils.

## Supporting information

S1 Table Clinical score: Spreadsheet showing Clinical arthritic scores recorded from 7–15 weeks of age. (XLSX)

S2 Table DXA analysis of femoral samples showing bone mineral density (BMD) and content (BMC) of 6-month-old mice. (XLSX)

S3 Table uCT parameters of TNF-a and TNF-GILZ Tg mice. (XLSX)

S4 Table ELISA assay of serum RANKL and OPG levels in the TNF-a and TNF-GILZ Tg mice. (XLSX)

S5 Table Multiplex immunoassay of cytokines and chemokines. (XLSX)

## References

[1] Hardy R, Cooper MS. Bone loss in inflammatory disorders. The Journal of endocrinology. 2009;201(3):309–20. doi: 10.1677/JOE-08-0568 . 19443863 10.1677/JOE-08-0568

[2] Redlich K, Smolen JS. Inflammatory bone loss: pathogenesis and therapeutic intervention. Nature reviews Drug discovery. 2012;11(3):234–50. Epub 2012/03/02. doi: 10.1038/nrd3669 . 22378270 10.1038/nrd3669

[3] Saxne T, Palladino MA, Heinegãrd D, Talal N, Wollheim FA. Detection of tumor necrosis factor α but not tumor necrosis factor β in rheumatoid arthritis synovial fluid and serum. Arthritis & Rheumatism. 1988;31(8):1041–5. 3136775 10.1002/art.1780310816

[4] Mateen S, Zafar A, Moin S, Khan AQ, Zubair S. Understanding the role of cytokines in the pathogenesis of rheumatoid arthritis. Clinica Chimica Acta. 2016;455:161–71. 10.1016/j.cca.2016.02.010 26883280

[5] Feldmann M. Development of anti-TNF therapy for rheumatoid arthritis. Nat Rev Immunol. 2002;2(5):364–71. doi: 10.1038/nri802 12033742 10.1038/nri802

[6] Roman-Blas JA, Jimenez SA. NF-Î°B as a potential therapeutic target in osteoarthritis and rheumatoid arthritis. Osteoarthritis and Cartilage. 2006;14(9):839–48. doi: 10.1016/j.joca.2006.04.008 16730463 10.1016/j.joca.2006.04.008

[7] Tsatsanis C, Androulidaki A, Venihaki M, Margioris AN. Signalling networks regulating cyclooxygenase-2. The International Journal of Biochemistry & Cell Biology. 2006;38(10):1654–61. 10.1016/j.biocel.2006.03.021 16713323

[8] Redlich K, Smolen JS. Inflammatory bone loss: pathogenesis and therapeutic intervention. Nat Rev Drug Discov. 2012;11(3):234–50. doi: 10.1038/nrd3669 22378270 10.1038/nrd3669

[9] Amarasekara DS, Yu J, Rho J. Bone Loss Triggered by the Cytokine Network in Inflammatory Autoimmune Diseases. Journal of immunology research. 2015:832127 doi: 10.1155/2015/832127 26065006 10.1155/2015/832127 PMC4434203

[10] De Benedetti F. The impact of chronic inflammation on the growing skeleton: lessons from interleukin-6 transgenic mice. Hormone research. 2009;72 Suppl 1:26–9. doi: 10.1159/000229760 . 19940492 10.1159/000229760

[11] Vis M, Havaardsholm EA, Haugeberg G, Uhlig T, Voskuyl AE, van de Stadt RJ, et al Evaluation of bone mineral density, bone metabolism, osteoprotegerin and receptor activator of the NFÎ°B ligand serum levels during treatment with infliximab in patients with rheumatoid arthritis. Ann Rheum Dis 2006 p. 1495–9. doi: 10.1136/ard.2005.044198 16606653 10.1136/ard.2005.044198 PMC1798341

[12] Siu S, Haraoui B, Bissonnette R, Bessette L, Roubille C, Richer V, et al Meta-Analysis of Tumor Necrosis Factor Inhibitors and Glucocorticoids on Bone Density in Rheumatoid Arthritis and Ankylosing Spondylitis Trials. Arthritis Care & Research. 2015;67(6):754–64. 25418272 10.1002/acr.22519

[13] Manara M, Sinigaglia L. Bone and TNF in rheumatoid arthritis: clinical implications. RMD Open. 2015;1(Suppl 1):e000065 doi: 10.1136/rmdopen-2015-000065 26557382 10.1136/rmdopen-2015-000065 PMC4632149

[14] Hoff M, Kvien TK, KÃ¤lvesten J, Elden A, Kavanaugh A, Haugeberg G. Adalimumab reduces hand bone loss in rheumatoid arthritis independent of clinical response: Subanalysis of the PREMIER study. BMC Musculoskeletal Disorders. 2011;12:54-. doi: 10.1186/1471-2474-12-54 21352592 10.1186/1471-2474-12-54 PMC3053306

[15] Finzel S, Rech J, Schmidt S, Engelke K, Englbrecht M, Stach C, et al Repair of bone erosions in rheumatoid arthritis treated with tumour necrosis factor inhibitors is based on bone apposition at the base of the erosion. Ann Rheum Dis 2011 p. 1587–93. doi: 10.1136/ard.2010.148395 21622765 10.1136/ard.2010.148395

[16] Raaschou P, Simard JF, Asker Hagelberg C, Askling J. Rheumatoid arthritis, anti-tumour necrosis factor treatment, and risk of squamous cell and basal cell skin cancer: cohort study based on nationwide prospectively recorded data from Sweden. BMJ 2016. 10.1136/bmj.i262 PMC4730989 26823527

[17] Singh JA, Wells GA, Christensen R, Ghogomu ET. Adverse effects of biologics: a network meta-analysis and Cochrane overview. The Cochrane database of systematic reviews. 2011;2. 10.1002/14651858.CD008794.pub2 PMC7173749 21328309

[18] Zhao Q, Hong D, Zhang Y, Sang Y. Association Between Anti-TNF Therapy for Rheumatoid Arthritis and Hypertension: A Meta-Analysis of Randomized Controlled Trials. Medicine. 2015;94(14). 10.1097/MD.0000000000000731 PMC4554042 25860222

[19] Setoguchi S, Schneeweiss S, Avorn J, Katz JN, Weinblatt ME, Levin R, et al Tumor necrosis factor-Î± antagonist use and heart failure in elderly patients with rheumatoid arthritis. American Heart Journal. 2008;156(2):336–41. doi: 10.1016/j.ahj.2008.02.025 18657665 10.1016/j.ahj.2008.02.025 PMC3257055

[20] Vultaggio A, Matucci A, Nencini F, Pratesi S, Parronchi P, Rossi O, et al Anti-infliximab IgE and non-IgE antibodies and induction of infusion-related severe anaphylactic reactions. Allergy. 2010;65(5):657–61. doi: 10.1111/j.1398-9995.2009.02280.x 19951375 10.1111/j.1398-9995.2009.02280.x

[21] Ghabril M, Bonkovsky HL, Kum C, Davern T, Hayashi PH, Kleiner DE, et al Liver Injury From Tumor Necrosis Factor-Î± Antagonists: Analysis of Thirty-four Cases. Clinical Gastroenterology and Hepatology. 2013;11(5):558–64.e3. doi: 10.1016/j.cgh.2012.12.025 23333219 10.1016/j.cgh.2012.12.025 PMC3865702

[22] Hastings R, Ding T, Butt S, Gadsby K, Zhang W, Moots RJ, et al Neutropenia in patients receiving anti–tumor necrosis factor therapy. Arthritis Care & Research. 2010;62(6):764–9. 20535786 10.1002/acr.20037

[23] Mohan N, Edwards E, Cupps T. Demyelination occurring during anti-tumor necrosis factor alpha therapy for inflammatory arthritides. Arthritis & Rheumatism. 2001;44(12):2862–9. 11762947 10.1002/1529-0131(200112)44:12<2862::aid-art474>3.0.co;2-w

[24] Clay M, Mazouyes A, M G. Risk of postoperative infections and the discontinuation of TNF inhibitors in patients with rheumatoid arthritis: A meta-analysis. Joint, bone, spine: revue du rhumatisme. 2016;Epub ahead of print. 10.1016/j.jbspin.2015.10.019 27118016

[25] Woodworth TG, den Broeder AA. Treating to target in established rheumatoid arthritis: Challenges and opportunities in an era of novel targeted therapies and biosimilars. Best Practice & Research Clinical Rheumatology. 2015;29(4–5):543–9. 26697765 10.1016/j.berh.2015.10.001

[26] van der Goes MC, Jacobs JW, Bijlsma JW. Rediscovering the therapeutic use of glucocorticoids in rheumatoid arthritis. Current opinion in rheumatology. 2016 doi: 10.1097/BOR.0000000000000278 26962704 10.1097/BOR.0000000000000278

[27] Hench PS, Kendall EC, Slocumb CH, Polley HF. The effect of a hormone of the adrenal cortex (17-hydroxy-11-dehydrocorticosterone: compound E) and of pituitary adrenocortical hormone in arthritis: preliminary report. Annals of the rheumatic diseases. 1949;8(2):97–104. 18623812 10.1136/ard.8.2.97 PMC1030685

[28] Kirwan JR, Bijlsma JW, Boers M, Shea BJ. Effects of glucocorticoids on radiological progression in rheumatoid arthritis. The Cochrane database of systematic reviews. 2007;(1):CD006356 doi: 10.1002/14651858.CD006356 . 17253590 10.1002/14651858.CD006356 PMC6465045

[29] Rousseau-Nepton I, Lang B, Rodd C. Long-term bone health in glucocorticoid-treated children with rheumatic diseases. Current rheumatology reports. 2013;15(3):315 doi: 10.1007/s11926-012-0315-x . 23358958 10.1007/s11926-012-0315-x

[30] Saag KG. Bone safety of low-dose glucocorticoids in rheumatic diseases. Annals of the New York Academy of Sciences. 2014;1318:55–64. doi: 10.1111/nyas.12446 . 24815076 10.1111/nyas.12446

[31] D'Adamio F, Zollo O, Moraca R, Ayroldi E, Bruscoli S, Bartoli A, et al A new dexamethasone-induced gene of the leucine zipper family protects T lymphocytes from TCR/CD3-activated cell death. Immunity. 1997;7(6):803–12. . 9430225 10.1016/s1074-7613(00)80398-2

[32] Yang N, Zhang W, Shi XM. Glucocorticoid-induced leucine zipper (GILZ) mediates glucocorticoid action and inhibits inflammatory cytokine-induced COX-2 expression. Journal of cellular biochemistry. 2008;103(6):1760–71. doi: 10.1002/jcb.21562 . 17910039 10.1002/jcb.21562

[33] Eddleston J, Herschbach J, Wagelie-Steffen AL, Christiansen SC, Zuraw BL. The anti-inflammatory effect of glucocorticoids is mediated by glucocorticoid-induced leucine zipper in epithelial cells. J Allergy Clin Immunol. 2007;119(1):115–22. doi: 10.1016/j.jaci.2006.08.027 17208592 10.1016/j.jaci.2006.08.027

[34] Beaulieu E, Ngo D, Santos L, Yang YH, Smith M, Jorgensen C, et al Glucocorticoid-induced leucine zipper is an endogenous antiinflammatory mediator in arthritis. Arthritis and rheumatism. 2010;62(9):2651–61. doi: 10.1002/art.27566 20496421 10.1002/art.27566

[35] Zhang W, Yang N, Shi XM. Regulation of mesenchymal stem cell osteogenic differentiation by glucocorticoid-induced leucine zipper (GILZ). The Journal of biological chemistry. 2008;283(8):4723–9. doi: 10.1074/jbc.M704147200 . 18084007 10.1074/jbc.M704147200

[36] He L, Yang N, Isales CM, Shi XM. Glucocorticoid-induced leucine zipper (GILZ) antagonizes TNF-alpha inhibition of mesenchymal stem cell osteogenic differentiation. PloS one. 2012;7(3):e31717 doi: 10.1371/journal.pone.0031717 ; PubMed Central PMCID: PMC3292550. 22396737 10.1371/journal.pone.0031717 PMC3292550

[37] Pan G, Cao J, Yang N, Ding K, Fan C, Xiong WC, et al Role of Glucocorticoid-induced Leucine Zipper (GILZ) in Bone Acquisition. The Journal of biological chemistry. 2014;289(28):19373–82. doi: 10.1074/jbc.M113.535237 ; PubMed Central PMCID: PMC4094049. 24860090 10.1074/jbc.M113.535237 PMC4094049

[38] Butler DM, Malfait AM, Mason LJ, Warden PJ, Kollias G, Maini RN, et al DBA/1 mice expressing the human TNF-alpha transgene develop a severe, erosive arthritis: characterization of the cytokine cascade and cellular composition. J Immunol 1997 p. 2867–76. 9300710

[39] Douni E, Sfikakis PP, Haralambous S, Fernandes P, Kollias G. Attenuation of inflammatory polyarthritis in TNF transgenic mice by diacerein: comparative analysis with dexamethasone, methotrexate and anti-TNF protocols. Arthritis Res Ther. 2004;6(1):R65–R72. doi: 10.1186/ar1028 14979939 10.1186/ar1028 PMC400419

[40] Armaka M, Gkretsi V, Kontoyiannis D, Kollias G. A standardized protocol for the isolation and culture of normal and arthritogenic murine synovial fibroblasts. 2009. 10.1038/nprot.2009.102.

[41] Keffer J, Probert L, Cazlaris H, Georgopoulos S, Kaslaris E, Kioussis D, et al Transgenic mice expressing human tumour necrosis factor: a predictive genetic model of arthritis. EMBO J. 1991;10(13):4025–31. 1721867 10.1002/j.1460-2075.1991.tb04978.x PMC453150

[42] Yang N, Baban B, Isales CM, Shi XM. Crosstalk between bone marrow-derived mesenchymal stem cells and regulatory T cells through a glucocorticoid-induced leucine zipper/developmental endothelial locus-1-dependent mechanism. FASEB journal: official publication of the Federation of American Societies for Experimental Biology. 2015;29(9):3954–63. Epub 2015/06/04. doi: 10.1096/fj.15-273664 . 26038125 10.1096/fj.15-273664 PMC4550369

[43] Szekanecz Z, Koch AE. Successes and failures of chemokine-pathway targeting in rheumatoid arthritis. Nature reviews Rheumatology. 2016;12(1):5–13. doi: 10.1038/nrrheum.2015.157 26607389 10.1038/nrrheum.2015.157

[44] Hampel U, Sesselmann S, Iserovich P, Sel S, Paulsen F, Sack R. Chemokine and cytokine levels in osteoarthritis and rheumatoid arthritis synovial fluid. Journal of Immunological Methods. 2013;396(1–2):134–9. doi: 10.1016/j.jim.2013.08.007 23994256 10.1016/j.jim.2013.08.007

[45] Caetano-Lopes J, Rodrigues A, Lopes A, Vale AC, Pitts-Kiefer MA, Vidal B, et al Rheumatoid Arthritis Bone Fragility Is Associated With Upregulation of IL17 and DKK1 Gene Expression. Clin Rev Allergy Immunol 2013 p. 38–45. 10.1007/s12016-013-8366-y 23546988

[46] Fine DH, Markowitz K, Fairlie K, Tischio-Bereski D, Ferrandiz J, Godboley D, et al Macrophage inflammatory protein-1alpha shows predictive value as a risk marker for subjects and sites vulnerable to bone loss in a longitudinal model of aggressive periodontitis. PloS one. 2014;9(6):e98541 doi: 10.1371/journal.pone.0098541 24901458 10.1371/journal.pone.0098541 PMC4047026

[47] Boström EA, Kindstedt E, Sulniute R, Palmqvist P, Majster M, Holm CK, et al Increased Eotaxin and MCP-1 Levels in Serum from Individuals with Periodontitis and in Human Gingival Fibroblasts Exposed to Pro-Inflammatory Cytokines. PloS one. 2015;10(8):e0134608 doi: 10.1371/journal.pone.0134608 26241961 10.1371/journal.pone.0134608 PMC4524692

[48] Li X, Makarov SS. An essential role of NF-kappaB in the "tumor-like" phenotype of arthritic synoviocytes. Proceedings of the National Academy of Sciences of the United States of America. 2006;103(46):17432–7. doi: 10.1073/pnas.0607939103 ; PubMed Central PMCID: PMC1859946. 17088540 10.1073/pnas.0607939103 PMC1859946

[49] Cenci S, Weitzmann MN, Roggia C, Namba N, Novack D, Woodring J, et al Estrogen deficiency induces bone loss by enhancing T-cell production of TNF-Î±. Journal of Clinical Investigation. 2000;106(10):1229–37. doi: 10.1172/JCI11066 11086024 10.1172/JCI11066 PMC381439

[50] Lam J, Takeshita S, Barker JE, Kanagawa O, Ross FP, Teitelbaum SL. TNF-Î± induces osteoclastogenesis by direct stimulation of macrophages exposed to permissive levels of RANK ligand. Journal of Clinical Investigation. 2000;106(12):1481–8. doi: 10.1172/JCI11176 11120755 10.1172/JCI11176 PMC387259

[51] Chopin F, Garnero P, le Henanff A, Debiais F, Daragon A, Roux C, et al Long-term effects of infliximab on bone and cartilage turnover markers in patients with rheumatoid arthritis. Ann Rheum Dis 2008 p. 353–7. doi: 10.1136/ard.2007.076604 17644538 10.1136/ard.2007.076604

[52] Krieckaert CLM, Nurmohamed MT, Wolbink G, Lems WF. Changes in bone mineral density during long-term treatment with adalimumab in patients with rheumatoid arthritis: a cohort study. Rheumatology (Oxford) 2013 p. 547–53. 10.1093/rheumatology/kes320 23221326

[53] van Everdingen AA, Jacobs JW, Siewertsz Van Reesema DR, Bijlsma JW. Low-dose prednisone therapy for patients with early active rheumatoid arthritis: clinical efficacy, disease-modifying properties, and side effects: a randomized, double-blind, placebo-controlled clinical trial. Annals of internal medicine. 2002;136(1):1–12. . 11777359 10.7326/0003-4819-136-1-200201010-00006

[54] Singh JA, Saag KG, Bridges SL Jr., Akl EA, Bannuru RR, Sullivan MC, et al 2015 American College of Rheumatology Guideline for the Treatment of Rheumatoid Arthritis. Arthritis & rheumatology (Hoboken, NJ. 2015;68(1):1–26. doi: 10.1002/art.39480 26545940 10.1002/art.39480

[55] Berrebi D, Bruscoli S, Cohen N, Foussat A, Migliorati G, Bouchet-Delbos L, et al Synthesis of glucocorticoid-induced leucine zipper (GILZ) by macrophages: an anti-inflammatory and immunosuppressive mechanism shared by glucocorticoids and IL-10. Blood. 2003;101(2):729–38. doi: 10.1182/blood-2002-02-0538 12393603 10.1182/blood-2002-02-0538

[56] Jorgensen C, Noel D, Gross G. Could inflammatory arthritis be triggered by progenitor cells in the joints? Ann Rheum Dis. 2002;61(1):6–9. ; PubMed Central PMCID: PMCPMC1753898. doi: 10.1136/ard.61.1.6 11779749 10.1136/ard.61.1.6 PMC1753898

[57] Jones EA, English A, Henshaw K, Kinsey SE, Markham AF, Emery P, et al Enumeration and phenotypic characterization of synovial fluid multipotential mesenchymal progenitor cells in inflammatory and degenerative arthritis. Arthritis and rheumatism. 2004;50(3):817–27. Epub 2004/03/17. doi: 10.1002/art.20203 . 15022324 10.1002/art.20203

[58] Li X, Makarov SS. An essential role of NF-kappaB in the "tumor-like" phenotype of arthritic synoviocytes. ProcNatlAcadSciUSA. 2006;103(46):17432–7. 10.1073/pnas.0607939103 PMC1859946 17088540

[59] Malysheva K, de Rooij K, LÃ¶wik CWGM, Baeten DL, Rose-John S, Stoika R, et al Interleukin 6/Wnt interactions in rheumatoid arthritis: interleukin 6 inhibits Wnt signaling in synovial fibroblasts and osteoblasts. Croatian Medical Journal. 2016;57(2):89–98. doi: 10.3325/cmj.2016.57.89 27106351 10.3325/cmj.2016.57.89 PMC4856197

